# Information Geometry of Randomized Quantum State Tomography

**DOI:** 10.3390/e20080609

**Published:** 2018-08-16

**Authors:** Akio Fujiwara, Koichi Yamagata

**Affiliations:** 1 Department of Mathematics, Osaka University, Toyonaka, Osaka 560-0043, Japan; 2 Graduate School of Informatics and Engineering, The University of Electro-Communications, Chofu, Tokyo 182-8585, Japan

**Keywords:** quantum state tomography, mutually unbiased bases, information geometry, dualistic foliation, mixed coordinate system

## Abstract

Suppose that a *d*-dimensional Hilbert space $H≃C^{d}$ admits a full set of mutually unbiased bases $\left\{|1^{\left(a\right)}\rangle ,\ldots ,|d^{\left(a\right)}\rangle \right\}$, where a=1,…,d+1. A randomized quantum state tomography is a scheme for estimating an unknown quantum state on H through iterative applications of measurements $M^{\left(a\right)}=\left\{|1^{\left(a\right)}\rangle \langle 1^{\left(a\right)}|,\ldots ,|d^{\left(a\right)}\rangle \langle d^{\left(a\right)}|\right\}$ for a=1,…,d+1, where the numbers of applications of these measurements are random variables. We show that the space of the resulting probability distributions enjoys a mutually orthogonal dualistic foliation structure, which provides us with a simple geometrical insight into the maximum likelihood method for the quantum state tomography.

## 1. Introduction

Quantum state tomography is a method of estimating an unknown quantum state represented on some Hilbert space H, consisting of a fixed set of measurements that provides sufficient information about the unknown quantum state, as well as a data processing that maps each measurement outcome into the quantum state space S(H) on H [1]. A set of measurements that fulfils this requirement is sometimes called a measurement basis. For mathematical simplicity, we restrict ourselves to Hilbert spaces of finite dimensions.

To elucidate our motivation, let us treat the simplest case when $H≃C^{2}$. It is well known that there is a one-to-one affine correspondence between the qubit state space

$$S\left(C^{2}\right):=\left\{\rho \in C^{2\times 2}\;|\;\rho \geq 0,\;\mathrm{Tr}\;\rho =1\right\}$$

and the unit ball (called the Bloch ball)

$$B:=\left.\left\{x=\left(x_{1},x_{2},x_{3}\right)\in R^{3}\;\right|\;{∥x∥}^{2}:={\left(x_{1}\right)}^{2}+{\left(x_{2}\right)}^{2}+{\left(x_{3}\right)}^{2}\leq 1\right\}.$$

In fact, the correspondence is explicitly given by the Stokes parametrization

$$x⟼\rho_{x}=\frac{1}{2}\left(I+x_{1}\sigma_{1}+x_{2}\sigma_{2}+x_{3}\sigma_{3}\right),$$

where $\sigma_{1}$, $\sigma_{2}$, and $\sigma_{3}$ are the standard Pauli matrices. Since $E_{\rho_{x}}\left[\sigma_{i}\right]:=\mathrm{Tr}\;\rho_{x}\sigma_{i}=x_{i}$ for i∈{1,2,3}, the set $\sigma =(\sigma_{1},\sigma_{2},\sigma_{3})$ of observables is regarded as an unbiased estimator [2,3,4] for the Stokes parameter $x=(x_{1},x_{2},x_{3})$. This is the basic idea behind the standard qubit state tomography, which runs as follows: suppose that, among *N* independent experiments, the *i*th Pauli matrix $\sigma_{i}$ was measured N/3 times, and outcomes +1 (spin-up) and −1 (spin-down) were obtained $n_{i}^{+}$ and $n_{i}^{-}$ times, respectively. Then a naive estimate for the true value of the parameter $x=(x_{1},x_{2},x_{3})$ is

$$\hat{x}=\left({\hat{x}}_{1},{\hat{x}}_{2},{\hat{x}}_{3}\right):=\left(\frac{n_{1}^{+}-n_{1}^{-}}{N/3},\;\frac{n_{2}^{+}-n_{2}^{-}}{N/3},\;\frac{n_{3}^{+}-n_{3}^{-}}{N/3}\right).$$

When the estimate $\hat{x}\in {\left[-1,1\right]}^{3}$ falls outside the Bloch ball *B*, it needs to be corrected so that the new estimate lies in the Bloch ball *B*. The maximum likelihood method is a canonical one to obtain a corrected estimate [2,5,6,7,8,9,10]. From the point of view of information geometry [11,12,13], the maximum likelihood estimate (MLE) is the orthogonal projection from the temporary estimate $\hat{x}$ onto the Bloch ball *B* with respect to the standard Fisher metric along the $\nabla^{\left(m\right)}$-geodesic [14], (cf., Appendix A).

Now let us deal with a slightly generalized situation: suppose that the *i*th Pauli matrix $\sigma_{i}$ was measured $N_{i}$ times and outcomes +1 and −1 were obtained $n_{i}^{+}$ and $n_{i}^{-}$ times, respectively, where ${\left\{N_{i}\right\}}_{i=1,2,3}$ were random variables. Such a situation arises in an actual experiment due to unexpected particle loss [15]. We shall call such a generalized estimation scheme a *randomized state tomography*. A naive estimate in this case is the following:

$$\hat{x}=\left({\hat{x}}_{1},{\hat{x}}_{2},{\hat{x}}_{3}\right):=\left(\frac{n_{1}^{+}-n_{1}^{-}}{N_{1}},\;\frac{n_{2}^{+}-n_{2}^{-}}{N_{2}},\;\frac{n_{3}^{+}-n_{3}^{-}}{N_{3}}\right).$$

One may invoke the maximum likelihood method when $\hat{x}$ falls outside the Bloch ball. It is then interesting to ask if there is also a useful geometrical picture for the MLE even when the numbers $N_{i}$ of measurements are random variables.

The above mentioned problem is naturally extended to quantum state tomography on an arbitrary Hilbert space that admits a full set of mutually unbiased bases [16,17]. In a *d*-dimensional Hilbert space $H≃C^{d}$, *k* orthonormal bases

$${\left\{|\alpha^{\left(1\right)}\rangle \right\}}_{\alpha \in \{1,\ldots ,d\}},{\left\{|\beta^{\left(2\right)}\rangle \right\}}_{\beta \in \{1,\ldots ,d\}},\ldots ,{\left\{|\gamma^{\left(k\right)}\rangle \right\}}_{\gamma \in \{1,\ldots ,d\}}$$

are called *mutually unbiased* if they satisfy

$${\left|\langle \alpha^{\left(a\right)}\;|\;\beta^{\left(b\right)}\rangle \right|}^{2}=\frac{1}{d}$$

for all a,b∈{1,…,k} with a≠b, and α,β∈{1,…,d}. It is known that the number *k* of mutually unbiased bases (MUBs) is at most d+1 [18]. If there are d+1 MUBs, the Hilbert space H is said to admit a full set of MUBs. For example, when the dimension *d* of H is a power of a prime, H admits a full set of MUBs [19]. Whether or not any Hilbert space admits a full set of MUBs is an open question [16].

In what follows, unless otherwise stated, we assume that the Hilbert space $H≃C^{d}$ under consideration admits a full set of MUBs. As demonstrated in Appendix B (cf., [17,20]), each density operator ρ∈S(H) can be uniquely represented as

(1) $$\rho =\rho \left(\xi \right):=\sum_{a=1}^{d+1}\left\{\sum_{\alpha =1}^{d-1}\xi_{\alpha }^{\left(a\right)}M_{\alpha }^{\left(a\right)}+\left(1-\sum_{\beta =1}^{d-1}\xi_{\beta }^{\left(a\right)}\right)M_{d}^{\left(a\right)}\right\}-I,$$

where

$$M^{\left(a\right)}:=\left\{M_{1}^{\left(a\right)},\ldots ,M_{d}^{\left(a\right)}\right\}=\left\{|1^{\left(a\right)}\rangle \langle 1^{\left(a\right)}|,\ldots ,|d^{\left(a\right)}\rangle \langle d^{\left(a\right)}|\right\}$$

is the projection-valued measure (PVM) associated with the *a*th orthogonal basis in the MUBs, and

$$\xi :={\left(\xi_{\alpha }^{\left(a\right)}\right)}_{\left(a,\alpha )\in \{1,\ldots ,d+1\}\times \{1,\ldots ,d-1\right\}}$$

is a $\left(d^{2}-1\right)$-dimensional real parameter that is chosen so that ρ(ξ)≥0. A simple calculation shows that, if the *a*th measurement $M^{\left(a\right)}$ is applied to the state ρ(ξ), one obtains each outcome α∈{1,…,d} with probability

(2) $$p_{\alpha }^{\left(a\right)}=\mathrm{Tr}\;\rho \left(\xi \right)M_{\alpha }^{\left(a\right)}=\left\{\begin{array}{cc} \xi_{\alpha }^{\left(a\right)}, & \mathrm{for}\;\alpha =1,\ldots ,d-1,\\ 1-\sum_{\beta =1}^{d-1}\xi_{\beta }^{\left(a\right)}, & \mathrm{for}\;\alpha =d. \end{array}\right.$$

This implies that the parametrization ξ↦ρ(ξ) establishes an affine isomorphism between the quantum state space

$$S\left(C^{d}\right):=\left\{\rho \in C^{d\times d}|\;\rho \geq 0,\;\mathrm{Tr}\;\rho =1\right\}$$

and the convex set

$$B:=\left\{\xi \in R^{d^{2}-1}|\;\rho \left(\xi \right)\geq 0\right\}.$$

Incidentally, the Stokes parametrization $x\mapsto \rho_{x}$ for the qubit state space $S(C^{2})$ is regarded as a special case of the above parametrization ξ↦ρ(ξ) for $S(C^{d})$. In fact, the eigenvectors of the Pauli matrices $\sigma_{1}$, $\sigma_{2}$, $\sigma_{3}$ form a full set of MUBs on $C^{2}$, and the Stokes parametrization $x=(x_{1},x_{2},x_{3})$ is related to the above parametrization $\xi =(\xi_{1}^{\left(1\right)},\xi_{1}^{\left(2\right)},\xi_{1}^{\left(3\right)})$ as

$$\xi_{1}^{\left(a\right)}=\frac{x_{a}+1}{2},\;\left(a=1,2,3\right).$$

Now that a standard affine parametrization ξ↦ρ(ξ) has been established on an arbitrary Hilbert space $H≃C^{d}$ that admits a full set of MUBs, the scheme of randomized state tomography is naturally extended to H as follows. Suppose that the *a*th measurement $M^{\left(a\right)}$ was applied $N^{\left(a\right)}$ times and the outcome α∈{1,…,d} was obtained $n_{\alpha }^{\left(a\right)}$ times, where ${\left\{N^{\left(a\right)}\right\}}_{a=1,\ldots ,d+1}$ were random variables. Then, due to (2), a naive estimate for the parameter $\xi_{\alpha }^{\left(a\right)}$ is

$${\hat{\xi }}_{\alpha }^{\left(a\right)}=\frac{n_{\alpha }^{\left(a\right)}}{N^{\left(a\right)}}.$$

When the estimate $\hat{\xi }:=\left({\hat{\xi }}_{\alpha }^{\left(a\right)}\right)\in {\left[0,1\right]}^{d^{2}-1}$ falls outside the parameter space *B*, one may invoke the maximum likelihood method to obtain a corrected estimate.

The objective of the present paper is to clarify that the $\nabla^{\left(m\right)}$-projection interpretation for the MLE is still valid for the randomized state tomography by changing the standard Fisher metric into a deformed one depending on the realization of the random variables $N^{\left(a\right)}$, which might as well be called a randomized Fisher metric. Such a novel geometrical picture will provide important insights into the quantum metrology.

The paper is organized as follows. In Section 2, we first introduce a statistical model on an extended sample space Ω that represents the randomized state tomography. We then clarify that the probability simplex P(Ω) is decomposed into mutually orthogonal dualistic foliation by means of certain $\nabla^{\left(m\right)}$- and $\nabla^{\left(e\right)}$-autoparallel submanifolds. In Section 3, we give a statistical interpretation of the above-mentioned dualistic foliation structure. In particular, we point out that the MLE is the $\nabla^{\left(m\right)}$-projection with respect to a deformed Fisher metric that depends on the realization of the random variables $N^{\left(a\right)}$. These results are demonstrated by several illustrative examples in Section 4. Finally, some concluding remarks are presented in Section 5. For the reader’s convenience, some background information is provided in Appendix A and Appendix B, including information geometry of the MLE and affine parametrization of a quantum state space S(H).

## 2. Geometry of Randomized State Tomography

We identify the randomized state tomography on $H≃C^{d}$ with the following scheme [21]: at each step of the measurement, one chooses a PVM $M^{\left(a\right)}$ at random with probability $s^{\left(a\right)}$, (a=1,…,d+1), and applies the chosen PVM to yield an outcome $\alpha \in \left\{1,\ldots ,d\right\}$. The sample space Ω for this statistical picture is

$$\Omega =\left\{(a,\alpha )|\;a\in \{1,\ldots ,d+1\},\;\alpha \in \{1,\ldots ,d\}\right\}.$$

Suppose that the unknown state ρ is specified by the coordinate ξ∈B as (1). Then the corresponding probability distribution on Ω is represented by the d(d+1)-dimensional probability vector

$$\begin{array}{c} p_{\left(s,\xi \right)}:=\left(s^{\left(1\right)}\left(\xi_{1}^{\left(1\right)},\ldots ,\xi_{d-1}^{\left(1\right)},1-\sum_{\alpha =1}^{d-1}\xi_{\alpha }^{\left(1\right)}\right),\ldots ,s^{\left(d\right)}\left(\xi_{1}^{\left(d\right)},\ldots ,\xi_{d-1}^{\left(d\right)},1-\sum_{\alpha =1}^{d-1}\xi_{\alpha }^{\left(d\right)}\right),\right.\\ \;\;\;\;\left.\left(1-\sum_{a=1}^{d}s^{\left(a\right)}\right)\left(\xi_{1}^{\left(d+1\right)},\ldots ,\xi_{d-1}^{\left(d+1\right)},1-\sum_{\alpha =1}^{d-1}\xi_{\alpha }^{\left(d+1\right)}\right)\right) \end{array}$$

where the parameter $s:=(s^{\left(1\right)},\ldots ,s^{\left(d\right)})$ belongs to the domain

$$D:=\left\{s\in R^{d}\;|\;s^{\left(a\right)}>0\;\mathrm{for}\;a\in \left\{1,\ldots ,d\right\},\;\;\mathrm{and}\;\sum_{a=1}^{d}s^{\left(a\right)}<1\right\}.$$

Note that the family

$$\left\{p_{\left(s,\xi \right)}|s\in D,\;\xi \in \Xi \right\}$$

with

Ξ:=ξ∈Rd2−1|ξα(a)>0for(a,α)∈{1,…,d+1}×{1,…,d−1},∑α=1d−1and∑α=1d−1ξα(a)<1fora∈{1,…,d+1}

forms a $\left(d^{2}+d-1\right)$-dimensional open probability simplex P(Ω), and the parameters (s,ξ) form a coordinate system of P(Ω). Since we are only interested in estimating the parameter ξ∈Ξ, the remaining parameter s∈D is understood as a set of nuisance parameters [2,12]. In what follows, we regard P(Ω) as a statistical manifold endowed with the standard dualistic structure $\left(g,\nabla^{\left(e\right)},\nabla^{\left(m\right)}\right)$, where *g* is the Fisher metric, and $\nabla^{\left(e\right)}$ and $\nabla^{\left(m\right)}$ are the exponential and mixture connections [12].

Let us consider the following submanifolds of P(Ω):

$$M\left(s\right):=\left\{p_{\left(s,\xi \right)}|\xi \in \Xi \right\}$$

for each s∈D, and

$$E\left(\xi \right):=\left\{p_{\left(s,\xi \right)}|s\in D\right\}$$

for each ξ∈Ξ. Since M(s) and E(ξ) are convex subsets of P(Ω), they are both $\nabla^{\left(m\right)}$-autoparallel. In addition, we have the following.

**Proposition** **1.** *For each ξ∈Ξ, the submanifold E(ξ) is $\nabla^{\left(e\right)}$-autoparallel. Furthermore, for each s∈D and ξ∈Ξ, the submanifolds M(s) and E(ξ) are mutually orthogonal with respect to the Fisher metric g.*

**Proof.**  Let us change the coordinate system (s,ξ) into $\left(\eta_{\langle a\rangle },\eta_{\langle b,\alpha \rangle }\right)$, where

$$\eta_{\langle a\rangle }:=s^{\left(a\right)}$$

for a∈{1,…,d}, and

$$\eta_{\langle b,\alpha \rangle }:=s^{\left(b\right)}\xi_{\alpha }^{\left(b\right)}$$

for (b,α)∈{1,…,d+1}×{1,…,d−1}. With this coordinate transformation, the probability vector $p_{\left(s,\xi \right)}$ is rewritten as

(3) $$p_{\eta }=\overset{d+1}{\underset{a=1}{⨁}}\left(\eta_{\langle a,1\rangle },\ldots ,\eta_{\langle a,d-1\rangle },\eta_{\langle a\rangle }-\sum_{\alpha =1}^{d-1}\eta_{\langle a,\alpha \rangle }\right).$$

Here, $\eta_{\langle d+1\rangle }$ is a function of ${\left\{\eta_{\langle a\rangle }\right\}}_{a\in \{1,\ldots ,d\}}$ defined by

$$\eta_{\langle d+1\rangle }:=1-\sum_{a=1}^{d}\eta_{\langle a\rangle },$$

and is not a component of the coordinate system $\eta :=(\eta_{\langle a\rangle },\eta_{\langle b,\alpha \rangle })$. We see from the representation (3) that the coordinate system η is $\nabla^{\left(m\right)}$-affine. The potential function for η is given by the negative entropy

$$\begin{array}{ccc} \varphi (\eta ) & := & \sum_{\omega \in \Omega }p_{\eta }\left(\omega \right)\log p_{\eta }\left(\omega \right)\\ & = & \sum_{a=1}^{d+1}\left\{\sum_{\alpha =1}^{d-1}\eta_{\langle a,\alpha \rangle }\log\eta_{\langle a,\alpha \rangle }+\left(\eta_{\langle a\rangle }-\sum_{\beta =1}^{d-1}\eta_{\langle a,\beta \rangle }\right)\log\left(\eta_{\langle a\rangle }-\sum_{\beta =1}^{d-1}\eta_{\langle a,\beta \rangle }\right)\right\} \end{array}$$

and the dual $\nabla^{\left(e\right)}$-affine coordinate system θ is given by

$$\theta^{\langle a\rangle }=\frac{\partial \varphi }{\partial \eta_{\langle a\rangle }}=\log\frac{s^{\left(a\right)}}{\left(1-\sum_{b=1}^{d}s^{\left(b\right)}\right)}+\log\frac{\left(1-\sum_{\beta =1}^{d-1}\xi_{\beta }^{\left(a\right)}\right)}{\left(1-\sum_{\beta =1}^{d-1}\xi_{\beta }^{\left(d+1\right)}\right)}$$

for a∈{1,…,d}, and

$$\theta^{\langle b,\alpha \rangle }=\frac{\partial \varphi }{\partial \eta_{\langle b,\alpha \rangle }}=\log\frac{\xi_{\alpha }^{\left(b\right)}}{\left(1-\sum_{\beta =1}^{d-1}\xi_{\beta }^{\left(b\right)}\right)}$$

for (b,α)∈{1,…,d+1}×{1,…,d−1}. Thus, fixing ξ is equivalent to fixing the coordinates ${\left(\theta^{\langle b,\alpha \rangle }\right)}_{\left(b,\alpha )\in \{1,\ldots ,d+1\}\times \{1,\ldots ,d-1\right\}}$, and the submanifold E(ξ) is generated by changing the remaining parameters ${\left(\theta^{\langle a\rangle }\right)}_{a\in \{1,\ldots ,d\}}$. This implies that E(ξ) is $\nabla^{\left(e\right)}$-autoparallel, proving the first part of the claim.

To prove the second part, let us introduce a mixed coordinate system [11]

$${\left(\eta_{\langle a\rangle };\theta^{\langle b,\alpha \rangle }\right)}_{a\in \{1,\ldots ,d\},\;(b,\alpha )\in \{1,\ldots ,d+1\}\times \{1,\ldots ,d-1\}}$$

of P(Ω). Since $\eta_{\langle a\rangle }=s^{\left(a\right)}$, the submanifold M(s) is rewritten as

$$M\left(s\right)=\left\{p_{\left(s,\xi \right)}|\;{\left(\eta_{\langle a\rangle }\right)}_{a\in \{1,\ldots ,d\}}\;\mathrm{are}\;\mathrm{fixed}\;\mathrm{and}\;{\left(\theta^{\langle b,\alpha \rangle }\right)}_{\left(b,\alpha )\in \{1,\ldots ,d+1\}\times \{1,\ldots ,d-1\right\}}\;\mathrm{are}\;\mathrm{arbitrary}\right\}.$$

On the other hand, the submanifold E(ξ) is rewritten as

$$E\left(\xi \right)=\left\{p_{\left(s,\xi \right)}|\;{\left(\theta^{\langle b,\alpha \rangle }\right)}_{\left(b,\alpha )\in \{1,\ldots ,d+1\}\times \{1,\ldots ,d-1\right\}}\;\mathrm{are}\;\mathrm{fixed}\;\mathrm{and}\;{\left(\eta_{\langle a\rangle }\right)}_{a\in \{1,\ldots ,d\}}\;\mathrm{are}\;\mathrm{arbitrary}\right\}.$$

Thus, the orthogonality of M(s) and E(ξ) is an immediate consequence of the orthogonality of the dual affine coordinate systems θ and η with respect to the Fisher metric *g*. ☐

**Figure 1:**
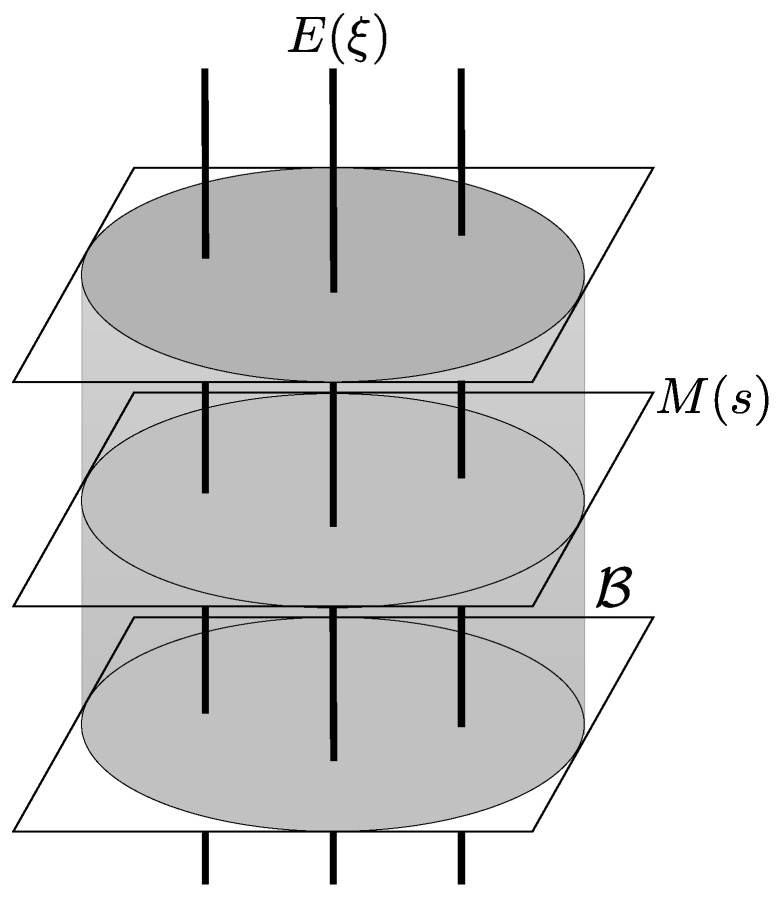
Mutually orthogonal dualistic foliation of P(Ω) based on M(s) and E(ξ). Each section M(s) is affinely isomorphic to the parameter space Ξ. The greyish cylindrical area indicates the subset $B=\{p_{\left(s,\xi \right)}|s\in D,\;\xi \in B\}$ of P(Ω). In particular, for each s∈D, the intersection M(s)∩B is affinely isomorphic to the physical domain *B* that corresponds to the state space S(H).

Proposition 1 implies that the manifold P(Ω) is decomposed into mutually orthogonal dualistic foliation based on the submanifolds M(s) and E(ξ), as illustrated in Figure 1. We shall exploit this geometrical structure in the next section.

## 3. Estimation of the Parameter ξ

Let us proceed to the problem of estimating the unknown parameter ξ using the randomized tomography. Suppose that, among *N* independent repetitions of experiments, the *a*th measurement $M^{\left(a\right)}$ was applied $N^{\left(a\right)}$ times and outcomes α∈{1,…,d} were obtained $n_{\alpha }^{\left(a\right)}$ times. Then temporary estimates $\left(\hat{s},\hat{\xi }\right)$ for the parameters (s,ξ) are given by

$${\hat{s}}^{\left(a\right)}:=\frac{N^{\left(a\right)}}{N}$$

for a∈{1,…,d}, and

$${\hat{\xi }}_{\beta }^{\left(b\right)}:=\frac{n_{\beta }^{\left(b\right)}}{N^{\left(b\right)}}$$

for (b,β)∈{1,…,d+1}×{1,…,d−1}. If $\hat{\xi }$ has fallen outside the physical domain *B*, one may seek a corrected estimate by the maximum likelihood method. Observe that, due to (2), the empirical distribution ${\hat{q}}_{N}\in P\left(\Omega \right)$ is represented as

(4) $${\hat{q}}_{N}=p_{\left(\hat{s},\hat{\xi }\right)}.$$

On the other hand, the physical domain *B* in the parameter space Ξ corresponds to the subset

$$B:=\{p_{\left(s,\xi \right)}\;|\;s\in D,\;\xi \in B\}$$

of P(Ω), (see Figure 1). The MLE $p^{*}$ in P(Ω) is then given by

(5) $$p^{*}=\underset{p\in B}{\arg\;\min}D\left({\hat{q}}_{N}∥p\right),$$

where D(·∥·) is the Kullback-Leibler divergence (cf., Appendix A). A crucial observation is the following.

**Proposition** **2.** *The minimum in (5) is achieved on $M(\hat{s})\cap B$.*

**Proof.**  Let us take a point $p_{\left(s,\xi \right)}\in B$ arbitrarily. It then follows from the mutually orthogonal dualistic foliation of P(Ω) established in Proposition 1 that

$$\begin{array}{ccc} D({\hat{q}}_{N}∥p_{\left(s,\xi \right)}) & = & D(p_{\left(\hat{s},\hat{\xi }\right)}∥p_{\left(s,\xi \right)})\\ & = & D\left(p_{\left(\hat{s},\hat{\xi }\right)}∥p_{\left(\hat{s},\xi \right)}\right)+D\left(p_{\left(\hat{s},\xi \right)}∥p_{\left(s,\xi \right)}\right)\\ & \geq & D(p_{\left(\hat{s},\hat{\xi }\right)}∥p_{\left(\hat{s},\xi \right)}). \end{array}$$

In the second equality, the generalized Pythagorean theorem was used. Consequently,

$$\min_{\xi \in B}D\left(p_{\left(\hat{s},\hat{\xi }\right)}∥p_{\left(s,\xi \right)}\right)\geq \min_{\xi \in B}D\left(p_{\left(\hat{s},\hat{\xi }\right)}∥p_{\left(\hat{s},\xi \right)}\right)$$

for all s∈D, and the right-hand side is achieved if and only if $s=\hat{s}$. ☐

**Figure 2:**
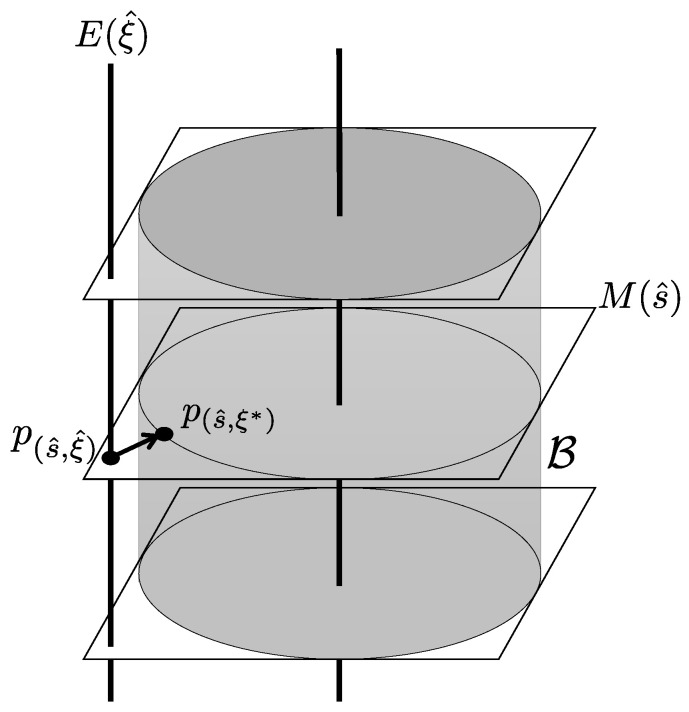
The maximum likelihood method in the framework of randomized tomography. Given a temporary estimate $\left(\hat{s},\hat{\xi }\right)$ with $\hat{\xi }\notin B$, we can restrict ourselves to the section $M(\hat{s})$ as the search space for the MLE $p^{*}$, and $p^{*}=p_{\left(\hat{s},\xi^{*}\right)}$ is the $\nabla^{\left(m\right)}$-projection from the empirical distribution $p_{\left(\hat{s},\hat{\xi }\right)}$ to B on the section $M(\hat{s})$.

The geometrical implication of Proposition 2 is illustrated in Figure 2. The MLE $p^{*}=p_{\left(\hat{s},\xi^{*}\right)}$ is the $\nabla^{\left(m\right)}$-projection from the empirical distribution $p_{\left(\hat{s},\hat{\xi }\right)}$ to B, and is on the section $M(\hat{s})$ specified by the temporary estimate $\hat{s}$.

Now we arrive at a geometrical picture behind the parameter estimation based on randomized state tomography. Suppose we are given a temporary estimate $\left(\hat{s},\hat{\xi }\right)$ with $\hat{\xi }\notin B$. Due to Proposition 2, we can restrict ourselves to section $M(\hat{s})$ as the search space for the MLE $p^{*}$. Since each section $M(\hat{s})$ is affinely isomorphic to the parameter space Ξ, we can introduce a dualistic structure $\left(\tilde{g},{\tilde{\nabla }}^{\left(e\right)},{\tilde{\nabla }}^{\left(m\right)}\right)$ on Ξ in the following way. Firstly, we identify the metric $\tilde{g}$ with the Fisher metric *g* restricted on $M(\hat{s})$, that is,

$$\begin{array}{ccc} {\tilde{g}}_{\left(\hat{s},\xi \right)}\left(\frac{\partial }{\partial \xi_{\alpha }^{\left(a\right)}},\frac{\partial }{\partial \xi_{\beta }^{\left(b\right)}}\right) & = & {\left.\frac{\partial \eta_{\langle a^{\prime },\alpha^{\prime }\rangle }}{\partial \xi_{\alpha }^{\left(a\right)}}\;\frac{\partial \eta_{\langle b^{\prime },\beta^{\prime }\rangle }}{\partial \xi_{\beta }^{\left(b\right)}}\;g_{\left(s,\xi \right)}\left(\frac{\partial }{\partial \eta_{\langle a^{\prime },\alpha^{\prime }\rangle }},\frac{\partial }{\partial \eta_{\langle b^{\prime },\beta^{\prime }\rangle }}\right)\right|}_{s=\hat{s}}\\ & = & {\left.s^{\left(a\right)}s^{\left(b\right)}\frac{\partial^{2}\varphi \left(\eta \right)}{\partial \eta_{\langle a,\alpha \rangle }\partial \eta_{\langle b,\beta \rangle }}\right|}_{s=\hat{s}}\\ & = & \delta_{ab}\;{\hat{s}}^{\left(a\right)}\left(\frac{1}{\xi_{d}^{\left(a\right)}}+\frac{\delta_{\alpha \beta }}{\xi_{\alpha }^{\left(a\right)}}\right), \end{array}$$

for a,b∈{1,…,d+1} and α,β∈{1,…,d−1}, where ${\hat{s}}^{\left(d+1\right)}$ and $\xi_{d}^{\left(a\right)}$ are formally defined as

$${\hat{s}}^{\left(d+1\right)}:=1-\sum_{a=1}^{d}{\hat{s}}^{\left(a\right)},\;\xi_{d}^{\left(a\right)}:=1-\sum_{\alpha =1}^{d-1}\xi_{\alpha }^{\left(a\right)}.$$

Secondly, the mixture connection ${\tilde{\nabla }}^{\left(m\right)}$ on Ξ is defined through the natural affine isomorphism between $M(\hat{s})$ and Ξ. Finally, the dual connection ${\tilde{\nabla }}^{\left(e\right)}$ is defined by the duality

$$\tilde{g}\left({\tilde{\nabla }}_{X}^{\left(e\right)}Y,Z\right):=X\tilde{g}\left(Y,Z\right)-\tilde{g}\left(Y,{\tilde{\nabla }}_{X}^{\left(m\right)}Z\right).$$

Thus, the MLE $\xi^{*}$ in the parameter space Ξ is interpreted as the ${\tilde{\nabla }}^{\left(m\right)}$-projection from $\hat{\xi }$ to the physical domain *B* with respect to the metric $\tilde{g}$.

## 4. Examples

In this section, we present some examples that demonstrate the implication of Proposition 2 as well as the general diagram given in Figure 2.

### 4.1. When dimH=2

Let us first study the simplest case when $H=C^{2}$. A full set of MUBs is given by

$$\begin{array}{c} \left\{|1^{\left(1\right)}\rangle ,|2^{\left(1\right)}\rangle \right\}=\left\{\frac{1}{\sqrt{2}}\left(\begin{array}{c} 1\\ 1 \end{array}\right),\frac{1}{\sqrt{2}}\left(\begin{array}{c} 1\\ -1 \end{array}\right)\right\},\\ \left\{|1^{\left(2\right)}\rangle ,|2^{\left(2\right)}\rangle \right\}=\left\{\frac{1}{\sqrt{2}}\left(\begin{array}{c} 1\\ -i \end{array}\right),\frac{1}{\sqrt{2}}\left(\begin{array}{c} 1\\ i \end{array}\right)\right\},\\ \left\{|1^{\left(3\right)}\rangle ,|2^{\left(3\right)}\rangle \right\}=\left\{\left(\begin{array}{c} 1\\ 0 \end{array}\right),\left(\begin{array}{c} 0\\ 1 \end{array}\right)\right\}. \end{array}$$

With these bases, the parameter representation (1) becomes

$$\rho =\frac{1}{2}\left(\begin{array}{cc} 1+x_{3} & x_{1}-ix_{2}\\ x_{1}+ix_{2} & 1-x_{3} \end{array}\right),$$

where $x=(x_{1},x_{2},x_{3})$ is the standard Stokes parameter, which is related to $\xi =(\xi_{1}^{\left(1\right)},\xi_{1}^{\left(2\right)},\xi_{1}^{\left(3\right)})$ as $x_{a}=2\xi_{1}^{\left(a\right)}-1$ for a=1,2,3.

**Figure 3:**
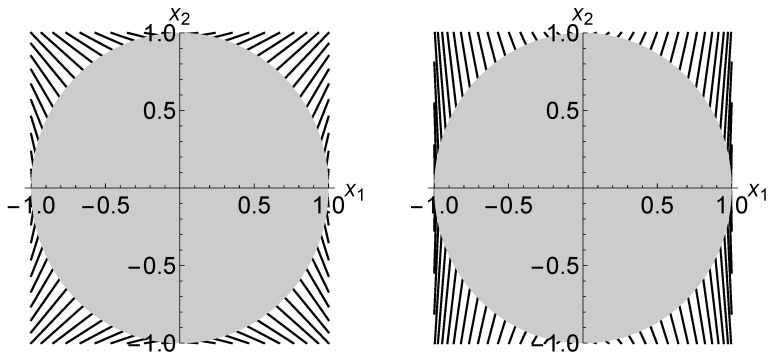
The trajectories of ${\tilde{\nabla }}^{\left(m\right)}$-projections on the Stokes parameter space when $N^{\left(1\right)}:N^{\left(2\right)}=1:1$ (**left**) and $N^{\left(1\right)}:N^{\left(2\right)}=5:1$ (**right**). The greyish disk represents the Bloch ball *B*.

Figure 3 demonstrates how the ${\tilde{\nabla }}^{\left(m\right)}$-projection is realized. Here, the trajectories of ${\tilde{\nabla }}^{\left(m\right)}$-projections that gives the MLE $p^{*}$ are plotted only on the $x_{1}x_{2}$-plane. The left and right panels correspond to the cases when $N^{\left(1\right)}:N^{\left(2\right)}=1:1$ and $N^{\left(1\right)}:N^{\left(2\right)}=5:1$, respectively. The change of $\xi_{1}$-coordinate relative to the change of $x_{2}$-coordinate along each trajectory is less noticeable in the right panel than in the left panel. This is because a tomography with $N^{\left(1\right)}/N^{\left(2\right)}=5$ provides us with more information about $x_{1}$-coordinate, relative to $x_{2}$-coordinate, as compared with the case when $N^{\left(1\right)}/N^{\left(2\right)}=1$.

### 4.2. When dimH=3

The space $H=C^{3}$ admits a full set of MUBs; for example,

$$\begin{array}{c} \left\{|1^{\left(1\right)}\rangle ,|2^{\left(1\right)}\rangle ,|3^{\left(1\right)}\rangle \right\}=\left\{\left(\begin{array}{c} 1\\ 0\\ 0 \end{array}\right),\left(\begin{array}{c} 0\\ 1\\ 0 \end{array}\right),\left(\begin{array}{c} 0\\ 0\\ 1 \end{array}\right)\right\},\\ \left\{|1^{\left(2\right)}\rangle ,|2^{\left(2\right)}\rangle ,|3^{\left(2\right)}\rangle \right\}=\left\{\frac{1}{\sqrt{3}}\left(\begin{array}{c} 1\\ 1\\ 1 \end{array}\right),\frac{1}{\sqrt{3}}\left(\begin{array}{c} 1\\ \omega \\ \omega^{2} \end{array}\right),\frac{1}{\sqrt{3}}\left(\begin{array}{c} 1\\ \omega^{2}\\ \omega \end{array}\right)\right\},\\ \left\{|1^{\left(3\right)}\rangle ,|2^{\left(3\right)}\rangle ,|3^{\left(3\right)}\rangle \right\}=\left\{\frac{1}{\sqrt{3}}\left(\begin{array}{c} \omega \\ 1\\ 1 \end{array}\right),\frac{1}{\sqrt{3}}\left(\begin{array}{c} 1\\ \omega \\ 1 \end{array}\right),\frac{1}{\sqrt{3}}\left(\begin{array}{c} 1\\ 1\\ \omega \end{array}\right)\right\},\\ \left\{|1^{\left(4\right)}\rangle ,|2^{\left(4\right)}\rangle ,|3^{\left(4\right)}\rangle \right\}=\left\{\frac{1}{\sqrt{3}}\left(\begin{array}{c} \omega^{2}\\ 1\\ 1 \end{array}\right),\frac{1}{\sqrt{3}}\left(\begin{array}{c} 1\\ \omega^{2}\\ 1 \end{array}\right),\frac{1}{\sqrt{3}}\left(\begin{array}{c} 1\\ 1\\ \omega^{2} \end{array}\right)\right\}, \end{array}$$

where $\omega =(-1+i\sqrt{3})/2$ is a primitive third root of unity. With these bases, the parameter representation (1) becomes

$$\rho =\left(\begin{array}{ccc} \xi_{1}^{\left(1\right)} & a_{12}-ib_{12} & a_{13}-ib_{13}\\ a_{12}+ib_{12} & \xi_{2}^{\left(1\right)} & a_{23}-ib_{23}\\ a_{13}+ib_{13} & a_{23}+ib_{23} & 1-\xi_{1}^{\left(1\right)}-\xi_{2}^{\left(1\right)} \end{array}\right),$$

where

$$\begin{array}{c} a_{12}=\frac{1}{2}\left(1+\xi_{1}^{\left(2\right)}-\xi_{1}^{\left(3\right)}-\xi_{2}^{\left(3\right)}-\xi_{1}^{\left(4\right)}-\xi_{2}^{\left(4\right)}\right),\\ a_{13}=\frac{1}{2}\left(-1+\xi_{1}^{\left(2\right)}+\xi_{2}^{\left(3\right)}+\xi_{2}^{\left(4\right)}\right),\\ a_{23}=\frac{1}{2}\left(-1+\xi_{1}^{\left(2\right)}+\xi_{1}^{\left(3\right)}+\xi_{1}^{\left(4\right)}\right),\\ b_{12}=\frac{\sqrt{3}}{6}\left(1-\xi_{1}^{\left(2\right)}-2\xi_{2}^{\left(2\right)}+\xi_{1}^{\left(3\right)}-\xi_{2}^{\left(3\right)}-\xi_{1}^{\left(4\right)}+\xi_{2}^{\left(4\right)}\right),\\ b_{13}=\frac{\sqrt{3}}{6}\left(-1+\xi_{1}^{\left(2\right)}+2\xi_{2}^{\left(2\right)}+2\xi_{1}^{\left(3\right)}+\xi_{2}^{\left(3\right)}-2\xi_{1}^{\left(4\right)}-\xi_{2}^{\left(4\right)}\right),\\ b_{23}=\frac{\sqrt{3}}{6}\left(1-\xi_{1}^{\left(2\right)}-2\xi_{2}^{\left(2\right)}+\xi_{1}^{\left(3\right)}+2\xi_{2}^{\left(3\right)}-\xi_{1}^{\left(4\right)}-2\xi_{2}^{\left(4\right)}\right). \end{array}$$

The physical domain *B* that corresponds to the state space $S(C^{3})$ is a compact convex subset of the parameter space $\Xi \;(\subset R^{8})$, and the extreme points of *B* form an algebraic variety with respect to the parameters

$$\xi =(\xi_{1}^{\left(1\right)},\xi_{2}^{\left(1\right)},\xi_{1}^{\left(2\right)},\xi_{2}^{\left(2\right)},\xi_{1}^{\left(3\right)},\xi_{2}^{\left(3\right)},\xi_{1}^{\left(4\right)},\xi_{2}^{\left(4\right)}).$$

**Figure 4:**
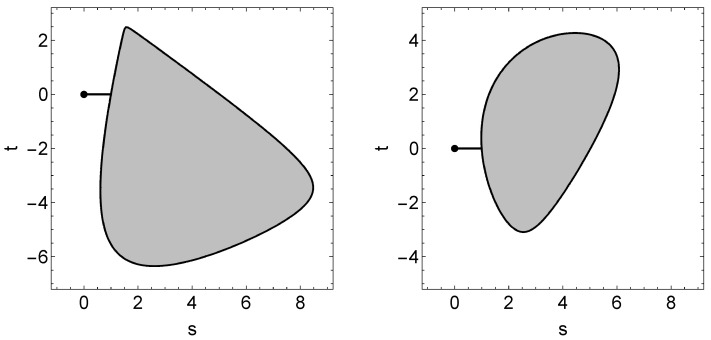
A trajectory of ${\tilde{\nabla }}^{\left(m\right)}$-projection displayed on randomly chosen two-dimensional affine subspaces of Ξ to which both the empirical distribution (marked as a dot) and the MLE belong. The greyish region represents the physical domain *B*.

A numerical example of a ${\tilde{\nabla }}^{\left(m\right)}$-projection that gives the MLE is illustrated in Figure 4, where no probe particle is lost, that is, when

$$\hat{s}=\left(\frac{1}{4},\frac{1}{4},\frac{1}{4},\frac{1}{4}\right).$$

In Figure 4, the dot laid outside the greyish region indicates the empirical distribution, i.e., the temporary estimate

$$\hat{\xi }=\left(0.100,0.100,0.066,0.333,0.333,0.333,0.333,0.333\right),$$

and the corresponding MLE is

$$\xi_{*}=\left(0.122,0.122,0.108,0.329,0.299,0.327,0.327,0.299\right).$$

Furthermore, the greyish region represents the physical domain *B* cut by a two-dimensional affine subspace of Ξ specified by the equation

$$\xi =\left(1-s\right)\;\hat{\xi }+s\;\xi_{*}+t\;v.$$

The vector *v* was chosen randomly under the condition that

$$v⊥\hat{\xi }-\xi_{*}\;\mathrm{and}\;∥v∥=∥\hat{\xi }-\xi_{*}∥,$$

where the orthogonality ⊥ and the norm ∥·∥ are understood relative to the standard Euclidean structure of $R^{8}$. In Figure 4, the vector *v* was taken to be

$$v_{1}=\left(-0.036,-0.038,0.012,-0.026,-0.038,0.011,0.002,0.005\right)$$

in the left panel, and

$$v_{2}=\left(0.028,0.000,-0.006,0.034,-0.024,-0.022,0.034,0.030\right)$$

in the right panel.

Figure 4 also demonstrates that the sections of the physical domain *B* show a variety of shapes. Unfortunately, due to this asymmetry of *B*, we were unable to find a (nontrivial) two-dimensional affine subspace on which every ${\tilde{\nabla }}^{\left(m\right)}$-projection runs. Such a difficulty is in good contrast to the simplest case $H≃C^{2}$, where the set *B* is rotationally symmetric and the ${\tilde{\nabla }}^{\left(m\right)}$-projections can be displayed on any two-dimensional section of *B* that passes through the origin of *B* as Figure 3.

### 4.3. When dimH≥4

The space $H=C^{4}$ is also known to admit a full set of MUBs since dimH=4 is the second power of the prime number 2; for example [22],

$$\begin{array}{c} \left\{|1^{\left(1\right)}\rangle ,|2^{\left(1\right)}\rangle ,|3^{\left(1\right)}\rangle ,|4^{\left(1\right)}\rangle \right\}=\left\{\left(\begin{array}{c} 1\\ 0\\ 0\\ 0 \end{array}\right),\left(\begin{array}{c} 0\\ 1\\ 0\\ 0 \end{array}\right),\left(\begin{array}{c} 0\\ 0\\ 1\\ 0 \end{array}\right),\left(\begin{array}{c} 0\\ 0\\ 0\\ 1 \end{array}\right)\right\},\\ \left\{|1^{\left(2\right)}\rangle ,|2^{\left(2\right)}\rangle ,|3^{\left(2\right)}\rangle ,|4^{\left(2\right)}\rangle \right\}=\left\{\frac{1}{2}\left(\begin{array}{c} 1\\ 1\\ 1\\ 1 \end{array}\right),\frac{1}{2}\left(\begin{array}{c} 1\\ 1\\ -1\\ -1 \end{array}\right),\frac{1}{2}\left(\begin{array}{c} 1\\ -1\\ -1\\ 1 \end{array}\right),\frac{1}{2}\left(\begin{array}{c} 1\\ -1\\ 1\\ -1 \end{array}\right)\right\},\\ \left\{|1^{\left(3\right)}\rangle ,|2^{\left(3\right)}\rangle ,|3^{\left(3\right)}\rangle ,|4^{\left(3\right)}\rangle \right\}=\left\{\frac{1}{2}\left(\begin{array}{c} 1\\ -1\\ -i\\ -i \end{array}\right),\frac{1}{2}\left(\begin{array}{c} 1\\ -1\\ i\\ i \end{array}\right),\frac{1}{2}\left(\begin{array}{c} 1\\ 1\\ i\\ -i \end{array}\right),\frac{1}{2}\left(\begin{array}{c} 1\\ 1\\ -i\\ i \end{array}\right)\right\},\\ \left\{|1^{\left(4\right)}\rangle ,|2^{\left(4\right)}\rangle ,|3^{\left(4\right)}\rangle ,|4^{\left(4\right)}\rangle \right\}=\left\{\frac{1}{2}\left(\begin{array}{c} 1\\ -i\\ -i\\ -1 \end{array}\right),\frac{1}{2}\left(\begin{array}{c} 1\\ -i\\ i\\ 1 \end{array}\right),\frac{1}{2}\left(\begin{array}{c} 1\\ i\\ i\\ -1 \end{array}\right),\frac{1}{2}\left(\begin{array}{c} 1\\ i\\ -i\\ 1 \end{array}\right)\right\},\\ \left\{|1^{\left(5\right)}\rangle ,|2^{\left(5\right)}\rangle ,|3^{\left(5\right)}\rangle ,|4^{\left(5\right)}\rangle \right\}=\left\{\frac{1}{2}\left(\begin{array}{c} 1\\ -i\\ -1\\ -i \end{array}\right),\frac{1}{2}\left(\begin{array}{c} 1\\ -i\\ 1\\ i \end{array}\right),\frac{1}{2}\left(\begin{array}{c} 1\\ i\\ -1\\ i \end{array}\right),\frac{1}{2}\left(\begin{array}{c} 1\\ i\\ 1\\ -i \end{array}\right)\right\}. \end{array}$$

It is straightforward to calculate the parameter representation (1) of a state $\rho \in S(C^{4})$; however, the corresponding density matrix is rather complicated, and we omit to display it here.

When $H=C^{6}$, or more generally, when dimH is not a power of a prime, we do not know whether H admits a full set of MUBs. Let us touch upon a situation where a Hilbert space H, if it exists, does not admit a full set of MUBs. In this case, there is no measurement basis $M^{\left(a\right)}$ that allows a parametrization ξ of the state space S(H) having a direct connection to the probability distribution of the outcomes as (2). Such a situation could be comparable to the case when the Gell-Mann matrices [23] are used as the measurement basis for estimating an unknown state on $H=C^{3}$. A state $\rho \in S(C^{3})$ is represented as

$$\rho =\rho_{x}:=\frac{1}{3}\left(I+\sqrt{3}\sum_{i=1}^{8}x_{i}\lambda_{i}\right),$$

where $\lambda_{1},\ldots ,\lambda_{8}$ are the Gell-Mann matrices, and $x=(x_{1},\ldots ,x_{8})$ is a set of real parameters. The physical domain

$$B=\left\{\left.x\in R^{8}\;\right|\;\rho_{x}\geq 0\right\}$$

forms a compact convex subset of the unit ball in $R^{8}$. With the state $\rho_{x}$, the probability distribution of obtaining the eigenvalues (−1,0,1) of the observable $\lambda_{1}$ is

$$\left(\frac{1-\sqrt{3}x_{1}+x_{8}}{3},\frac{1-2x_{8}}{3},\frac{1+\sqrt{3}x_{1}+x_{8}}{3}\right),$$

while the probability distribution of obtaining the eigenvalues (−1,0,1) of the observable $\lambda_{2}$ is

$$\left(\frac{1-\sqrt{3}x_{2}+x_{8}}{3},\frac{1-2x_{8}}{3},\frac{1+\sqrt{3}x_{2}+x_{8}}{3}\right).$$

Note that the probability of obtaining the eigenvalue 0 of $\lambda_{1}$ is identical to that of $\lambda_{2}$. However, in a randomized estimation scheme in which $\lambda_{i}$ is measured $N_{i}$ times, the frequency of obtaining the eigenvalue 0 of $\lambda_{1}$ would be different from that of $\lambda_{2}$. Consequently, one cannot assign a consistent temporary estimate ${\hat{x}}_{8}$ for the parameter $x_{8}$ in that case. Put differently, the empirical distribution ${\hat{q}}_{N}$ on the extended outcome space Ω does not in general have a coordinate representation (4). Thus, the existence of a full set of MUBs is crucial in our analysis.

## 5. Concluding Remarks

In the present paper, we explored an information geometrical structure of the randomized quantum state tomography, assuming that the Hilbert space under consideration admits a full set of MUBs. We first introduced a classical statistical model ${\left\{p_{\left(s,\xi \right)}\right\}}_{s,\xi }$ on an extended sample space Ω, and found that the probability simplex P(Ω) was decomposed into mutually orthogonal dualistic foliation (Proposition 1). We then clarified that this geometrical structure had a statistical importance in estimating the coordinate ξ of an unknown quantum state ρ(ξ) under the existence of the nuisance parameter *s* (Proposition 2). This result gave a generalized insight into the $\nabla^{\left(m\right)}$-projection interpretation for the MLE in that a similar interpretation was still valid for the randomized quantum state tomography by changing the standard Fisher metric into a deformed one. It also provided us with a new, convenient way of data processing in the actual quantum state tomography that may involve unexpected probe particle loss.

It should be noted that the existence of a full set of MUBs ensures the parametrization (1) of the quantum state space S(H). Such a parametrization is distinctive in that it enables a direct correspondence between the parameter space and the probability simplex, realizing the coordinate representation (4) of the empirical distribution ${\hat{q}}_{N}$. Thus, the use of a full set of MUBs is crucial in our analysis. Nevertheless, it is often the case that the Hilbert space under consideration takes the form $H≃{\left(C^{p}\right)}^{⊗n}$ for p=2 or 3 because qubits or qutrits are often regarded as building blocks of various quantum protocols. Therefore, the existence of a full set of MUBs would not be too strong a requirement in applications.

## Figures and Tables

**Figure A1:**
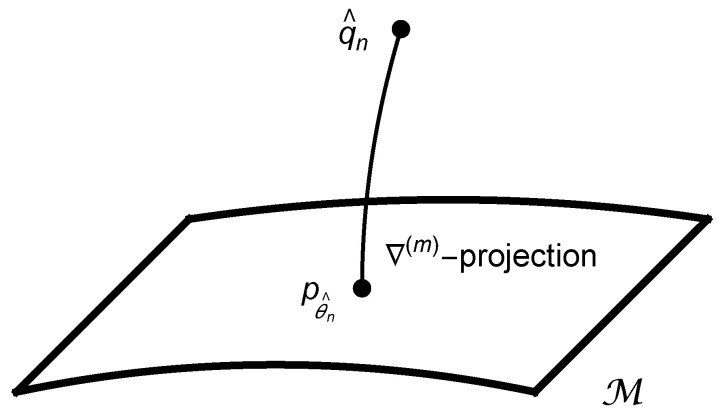
The maximum likelihood estimate $p_{{\hat{\theta }}_{n}}$ is the minimizer of the function $p\mapsto D({\hat{q}}_{n}∥p)$ with respect to p∈M, and is also understood as the $\nabla^{\left(m\right)}$-projection from the empirical distribution ${\hat{q}}_{n}$ to M or its boundary.

## Abbreviations

The following abbreviations are used in this manuscript:

MUBs	mutually unbiased bases
PVM	projection-valued measure
MLE	maximum likelihood estimate

## Appendix A. Information Geometry of MLE

Let P(Ω) denote the set of probability distributions on a finite sample space Ω, i.e.,

$$P\left(\Omega \right):=\left\{p:\Omega \to R\;\left|\;p\left(\omega \right)>0\mathrm{for}\mathrm{all}\omega \in \Omega ,\mathrm{and}\sum_{\omega \in \Omega }p\left(\omega \right)=1\right\}\right..$$

This set may be identified with the (|Ω|−1)-dimensional (open) simplex, where |Ω| denotes the number of elements in Ω, and thus it is sometimes referred to as the *probability simplex* on Ω. The set P(Ω) is also regarded as a *statistical manifold* endowed with the *dualistic structure* $\left(g,\nabla^{\left(e\right)},\nabla^{\left(m\right)}\right)$, where *g* is the *Fisher metric*, and $\nabla^{\left(e\right)}$ and $\nabla^{\left(m\right)}$ are the *exponential* and *mixture connections* [11,12,13].

Suppose that the state of the physical system at hand belongs to a (closed) subset M of P(Ω), but we do not know which is the true state. We further assume that the probability distributions of M are faithfully parametrized by a finite dimensional parameter θ as

$$M=\{p_{\theta }\left(\omega \right)\;|\;\theta \in \Theta \}.$$

In this case, M is called a *parametric model*, and our task is to estimate the true value of the parameter θ that specifies the true state. Suppose that, by *n* independent experiments, we have obtained data $\left(x_{1},x_{2},\ldots ,x_{n}\right)\in \Omega^{n}$. This information is compressed into the *empirical distribution*, an element of P(Ω) defined by

$$\begin{array}{ccc} {\hat{q}}_{n}\left(\omega \right) & := & \frac{\mathrm{Number}\;\mathrm{of}\;\mathrm{occurrences}\;\mathrm{of}\;\omega \;\mathrm{in}\;\mathrm{data}\;(x_{1},x_{2},\ldots ,x_{n})}{n}\\ & = & \frac{1}{n}\sum_{i=1}^{n}\delta_{x_{i}}\left(\omega \right) \end{array}$$

for each ω∈Ω, where $\delta_{x_{i}}\left(\omega \right)$ is the Kronecker delta. If ${\hat{q}}_{n}$ belongs to the model M, then we have an estimate ${\hat{\theta }}_{n}$ that satisfies $p_{{\hat{\theta }}_{n}}={\hat{q}}_{n}$. However, the empirical distribution ${\hat{q}}_{n}$ does not always belong to the model M. When ${\hat{q}}_{n}\notin M$, we need to find an alternative estimate from the data. A canonical method of finding an alternative estimate $p_{{\hat{\theta }}_{n}}\in M$ is the *maximum likelihood method*, in which one seeks the maximizer of the likelihood function

$$\theta ⟼p_{\theta }\left(x_{1}\right)p_{\theta }\left(x_{2}\right)\ldots p_{\theta }\left(x_{n}\right),$$

within the domain Θ of the parameter θ, so that

$${\hat{\theta }}_{n}:=\underset{\theta \in \Theta }{\arg\;\max}\left\{p_{\theta }\left(x_{1}\right)p_{\theta }\left(x_{2}\right)\ldots p_{\theta }\left(x_{n}\right)\right\}.$$

We can rewrite this relation as follows.

$$\begin{array}{ccc} {\hat{\theta }}_{n} & = & \underset{\theta \in \Theta }{\arg\;\max}\;\frac{1}{n}\sum_{i=1}^{n}\log p_{\theta }\left(x_{i}\right)\\ & = & \underset{\theta \in \Theta }{\arg\;\max}\;\sum_{\omega \in \Omega }{\hat{q}}_{n}\left(\omega \right)\log p_{\theta }\left(\omega \right)\\ & = & \underset{\theta \in \Theta }{\arg\;\min}\;\sum_{\omega \in \Omega }{\hat{q}}_{n}\left(\omega \right)\left\{\log{\hat{q}}_{n}\left(\omega \right)-\log p_{\theta }\left(\omega \right)\right\}\\ & = & \underset{\theta \in \Theta }{\arg\;\min}\;D\left({\hat{q}}_{n}∥p_{\theta }\right), \end{array}$$

where

$$D\left(q∥p\right):=\sum_{\omega \in \Omega }q\left(\omega \right)\log\frac{q(\omega )}{p(\omega )}$$

is the *Kullback-Leibler divergence* from *q* to *p*. In other words, the maximum likelihood estimate (MLE) $p_{{\hat{\theta }}_{n}}$ is the point on M that is “closest” from the empirical distribution ${\hat{q}}_{n}$ as measured by the Kullback-Leibler divergence:

$$p_{{\hat{\theta }}_{n}}=\underset{p\in M}{\arg\;\min}\;D\left({\hat{q}}_{n}∥p\right).$$

Due to the generalized Pythagorean theorem, the MLE is geometrically understood as the $\nabla^{\left(m\right)}$-projection from ${\hat{q}}_{n}$ to M or its boundary, as illustrated in Figure A1.

## Appendix B. Parametrization of S(H)

Suppose that the Hilbert space $H≃C^{d}$ under consideration admits a full set of MUBs

$${\left\{|\alpha^{\left(a\right)}\rangle \right\}}_{\alpha \in \{1,\ldots ,d\}},\;\left(a=1,\ldots ,d+1\right).$$

For each a∈{1,…,d+1}, let

$$M^{\left(a\right)}:=\left\{M_{1}^{\left(a\right)},\ldots ,M_{d}^{\left(a\right)}\right\}=\left\{|1^{\left(a\right)}\rangle \langle 1^{\left(a\right)}|,\ldots ,|d^{\left(a\right)}\rangle \langle d^{\left(a\right)}|\right\}.$$

Then, the operators

$${\left\{M_{\alpha }^{\left(a\right)}-\frac{I}{d}\right\}}_{\left(a,\alpha )\in \{1,\ldots ,d+1\}\times \{1,\ldots ,d-1\right\}}$$

are linearly independent, spanning the space of selfadjoint operators with zero trace. This is easily seen from the orthogonality relation:

$$\mathrm{Tr}\;\left(M_{\alpha }^{\left(a\right)}-\frac{I}{d}\right)\left(M_{\beta }^{\left(b\right)}-\frac{I}{d}\right)=\delta_{ab}\left(\delta_{\alpha \beta }-\frac{1}{d}\right).$$

Thus, given ρ∈S(H), the operator ρ−(I/d) is uniquely expanded as

$$\rho -\frac{I}{d}=\sum_{a=1}^{d+1}\sum_{\alpha =1}^{d-1}x_{\alpha }^{\left(a\right)}\left(M_{\alpha }^{\left(a\right)}-\frac{I}{d}\right),$$

where $x_{\alpha }^{\left(a\right)}$ are real numbers. We can regard $x_{\alpha }^{\left(a\right)}$ as a coordinate system of the state space S(H). When d=2, this is identical to the Stokes parametrization, up to a factor of 2.

Now, let us change the coordinate system $x_{\alpha }^{\left(a\right)}$ into $\xi_{\alpha }^{\left(a\right)}$ as

$$x_{\alpha }^{\left(a\right)}=\xi_{\alpha }^{\left(a\right)}+\left(\sum_{\beta =1}^{d-1}\xi_{\beta }^{\left(a\right)}\right)-1.$$

We then arrive at the parametrization (1), i.e.,

$$\rho =\sum_{a=1}^{d+1}\left\{\sum_{\alpha =1}^{d-1}\xi_{\alpha }^{\left(a\right)}M_{\alpha }^{\left(a\right)}+\left(1-\sum_{\beta =1}^{d-1}\xi_{\beta }^{\left(a\right)}\right)M_{d}^{\left(a\right)}\right\}-I.$$

This parametrization is useful in our analysis because it gives a direct connection to the probability distribution of outcomes of the measurement $M^{\left(a\right)}$ as

$$p_{\alpha }^{\left(a\right)}:=\mathrm{Tr}\;\rho M_{\alpha }^{\left(a\right)}=\left\{\begin{array}{cc} \xi_{\alpha }^{\left(a\right)}, & \mathrm{for}\;\alpha =1,\ldots ,d-1,\\ 1-\sum_{\beta =1}^{d-1}\xi_{\beta }^{\left(a\right)}, & \mathrm{for}\;\alpha =d. \end{array}\right.$$
